# Robotic versus laparoscopic inguinal hernia repair: a systematic review and meta-analysis of randomized controlled trials

**DOI:** 10.1007/s00464-026-13253-y

**Published:** 2026-08-20

**Authors:** Carlo Alberto Schena, Vito Laterza, Fausto Rosa

**Affiliations:** 1 Department of General Surgery, Fondazione Poliambulanza Istituto Ospedaliero, Via Leonida Bissolati, 57, 25124 Brescia, BS Italy; 2 Department of Digestive Surgery, Surgical Oncology and Liver Transplantation, University Hospital of Besançon, Besançon, France; 3 Department of Translational Medicine and Surgery, Università Cattolica del Sacro Cuore Facoltà di Medicina e Chirurgia, Largo Francesco Vito, 1, 00168 Rome, RM Italy; 4 Department of Digestive Surgery, Fondazione Policlinico Universitario Gemelli IRCCS, Rome, Italy

**Keywords:** Inguinal hernia, Robotic surgery, Laparoscopic surgery, Systematic review, Meta-analysis, Randomized controlled trials, Minimally invasive hernia repair, GRADE

## Abstract

**Background:**

Robotic inguinal hernia repair has experienced rapid adoption despite limited high-quality comparative evidence. Previous meta-analyses have mainly focused on non-randomized series, and the only synthesis restricted to RCTs included only three trials and short-term outcomes. This systematic review and meta-analysis synthesizes all available RCT evidence across clinical, patient-reported, and surgeon-centered endpoints.

**Methods:**

A systematic search of MEDLINE, Embase, Scopus, Web of Science, and Cochrane CENTRAL was performed. RCTs comparing robotic versus laparoscopic inguinal hernia repair in adults were eligible. Primary outcomes included overall postoperative complications, hernia recurrence, and same-day discharge rate. Secondary outcomes encompassed operative time, chronic postoperative inguinal pain, estimated blood loss, seroma, hematoma, urinary retention, readmission, quality of life, surgeon ergonomics, surgical inflammatory response, stray thermal energy, and procedural cost. Pooled risk ratios (RR), risk difference (RD), or mean differences (MD) with 95% confidence intervals were calculated using the Mantel–Haenszel or inverse-variance method. Heterogeneity was evaluated with the *I*^2^ statistic. Risk of bias was assessed using the Cochrane RoB 2 tool, and the certainty of evidence was graded with GRADE.

**Results:**

Seven RCTs reported in nine publications (691 patients) were included. No statistically significant differences were found in overall postoperative complications (RR 0.76, 95% CI 0.49–1.17; *I*^2^ = 14%; *p* = 0.22), hernia recurrence (RD − 0.00, 95% CI − 0.02 to 0.02; *I*^2^ = 0%; *p* = 1.00), or same-day discharge rate (RR 1.02, 95% CI 0.93–1.13; *I*^2^ = 60%; *p* = 0.66). All secondary pooled analyses were non-significant. Acute pain could not be pooled due to heterogeneous measurement scales and timepoints across trials. The certainty of evidence was rated low to very low across all outcomes.

**Conclusions:**

No statistically significant differences were found between robotic and laparoscopic inguinal hernia repair for any primary or secondary outcome, with low to very low certainty for all endpoints.

**Supplementary Information:**

The online version contains supplementary material available at https://doi.org/10.1007/s00464-026-13253-y.

Every 40 s, somewhere in the world, a surgeon repairs an inguinal hernia, making it one of the most common surgeries in medicine, with an estimated 20 million cases annually [1, 2]. For decades, the main debate focused on open versus laparoscopic repair; that question is now mostly settled in favor of minimally invasive techniques for elective cases, which have lower rates of chronic postoperative inguinal pain, quicker recovery, and similar recurrence rates compared with the Lichtenstein procedure [3–5]. The focus has shifted: today, the question is whether adding a robot to an established laparoscopic procedure offers meaningful benefits for patients, surgeons, or health systems. In the United States Veterans Affairs system alone, robotic inguinal hernia repairs rose from 0.24 to 19.6%. 6% between 2008 and 2019, a nearly 80-fold increase driven mainly by institutional investment, industry promotion, and surgeon adoption, rather than solid evidence [6]. The claimed benefits of robotic systems (such as tremor filtration, wristed articulation, 3D view, and ergonomic console design) are mechanistically understandable. However, whether these translate into measurable patient benefits and whether such benefits justify costs that are consistently two to three times higher than laparoscopy remain open questions. Past systematic reviews and meta-analyses comparing robotic and laparoscopic inguinal hernia repair have shown conflicting results; their limitations are well known: many rely on non-randomized, retrospective, single-surgeon series, which are complicated by learning curves, case mix, and institutional volume effects that are difficult to disentangle post hoc [7–9]. Additionally, variability in outcome measures, such as different pain scales, inconsistent operative time definitions, and non-standardized complication classifications, further complicates interpretation and emphasizes the need for analyses that explicitly evaluate research validity across studies [10]. The only prior meta-analysis restricted to randomized controlled trials (RCTs) included just three studies and focused on short-term outcomes [11], leaving a gap in the broader evidence base. Since then, new RCTs have been published reporting outcomes on diverse topics such as systemic inflammatory response, thermal energy spread, surgeon cognitive load, ergonomic risk, and quality of life, areas that previous summaries have not attempted to synthesize.

This systematic review and meta-analysis addresses this gap by synthesizing the full available RCT evidence across clinical, patient-reported, and surgeon-centered outcomes, with clear focus on the methodological sources of heterogeneity that have limited earlier analyses.

## Materials and methods

### Study design and reporting

This systematic review and meta-analysis was reported in accordance with the Preferred Reporting Items for Systematic Reviews and Meta-Analyses (PRISMA 2020) Statement [12] (see eTable 1 for the PRISMA 2020 checklist). The review protocol, including eligibility criteria, search strategy, data extraction, and analysis plans, was defined a priori and prospectively registered on the International Prospective Register of Ongoing Systematic Reviews (PROSPERO) under the registration number CRD420261370145. The study aimed to summarize the highest quality comparative evidence by including only RCTs that evaluate robotic versus laparoscopic minimally invasive inguinal hernia repair.

### Eligibility criteria

*Types of participants:* Eligible studies included adult patients (≥ 18 years) undergoing minimally invasive repair of inguinal hernia. Trials involving unilateral or bilateral hernias, primary or recurrent hernias, and various hernia classifications were eligible.

*Types of interventions and comparators* The intervention of interest was robotic minimally invasive inguinal hernia repair, including robotic transabdominal preperitoneal (r-TAPP) repair and robotic totally extraperitoneal (r-TEP) repair when reported. The comparator was conventional laparoscopic minimally invasive inguinal hernia repair, including laparoscopic transabdominal preperitoneal (l-TAPP) and laparoscopic totally extraperitoneal (l-TEP) repair. Trials comparing robotic or laparoscopic repair to open surgery only were not eligible.

*Outcomes* The primary outcomes of interest included overall postoperative complications, hernia recurrence at the longest available follow-up, same-day discharge rate, and postoperative pain (as reported by each trial, usually measured using a visual analogue scale or numeric rating scale at a specific postoperative time point). Additional outcomes encompassed chronic pain at the longest follow-up, operative time, readmission rates, seroma, hematoma, urinary retention, time to resume normal activity or work, conversion to open surgery, surgical site infection, patient-reported outcomes measures, including quality-of-life scores when available, patient-reported experience measures when reported. Other measures included surgical workload and ergonomics, perioperative inflammatory response, and cost-related endpoints when available. When outcomes were reported at multiple time points, acute outcomes were recorded at the earliest clinically relevant assessment, and chronic pain or recurrence at the longest follow-up. Multiple publications from the same trial were consolidated as separate timeframes of the same randomized cohort.

*Types of studies* RCTs comparing robotic and laparoscopic minimally invasive inguinal hernia repair in adults were considered eligible. Studies including unilateral or bilateral hernias, primary or recurrent hernias, and either TAPP or TEP repair were eligible, as long as robotic and laparoscopic techniques were directly compared within the same randomized design. Trials published as full-text articles or conference papers with enough extractable data were eligible. Quasi-randomized studies, non-randomized comparative studies, retrospective cohorts, case series, editorials, narrative reviews, letters without original data, and conference abstracts lacking sufficient data were excluded.

*Setting and context* No restrictions were imposed on healthcare settings (such as academic or community hospitals, high- or low-volume centers) or geographical regions. Trials in any country and clinical setting were eligible.

### Literature search strategy

A comprehensive literature search was conducted across four electronic databases: MEDLINE (via PubMed), Embase, Scopus, and Web of Science. Additionally, the Cochrane Central Register of Controlled Trials (CENTRAL) was searched for randomized trials. The search strategy was customized for each database and combined controlled vocabulary terms with free-text keywords related to inguinal hernia, robotic surgery, and laparoscopy. No restrictions were placed on language or publication year, and studies in non-English languages were eligible if sufficient information was available for assessment and extraction. Reference lists from all included studies and relevant reviews were hand-searched to find extra potential trials. When feasible, clinical trial registries were also screened for ongoing or completed but unpublished trials. Sample strategies for CENTRAL, Scopus, Embase, and Web of Science are included in the Supplementary Material (eTable 2). The searches were last updated on April 13, 2026.

### Study selection

All references identified through electronic searches were exported to a citation management software (EndNote 2025, Clarivate Analytics, Philadelphia, PA, USA) and deduplicated before screening. After removing duplicate records, two reviewers (FR, VL) independently and blindly screened titles and abstracts for potential eligibility. To maximize sensitivity, records were excluded at this stage only when both reviewers agreed that the study did not meet the inclusion criteria. Full texts of potentially relevant articles were then assessed independently by the same reviewers. Disagreements at any stage were resolved through discussion and, when necessary, by consulting a third reviewer (CAS). When multiple reports referred to the same randomized trial, these publications were combined and treated as a single study, with the most complete and mature dataset used for each outcome and time point.

### Data extraction

Data were independently extracted by two reviewers (VL, CAS) using a predefined standardized extraction form. The data collection involved a standardized form that captured study and patient characteristics, details of surgical techniques, surgeon experience requirements, follow-up durations, all pre-specified outcomes with measures of variability, and funding sources. Outcome data were separately extracted for robotic and laparoscopic groups. For binary variables, the number of events and the number of patients at risk were recorded. For continuous outcomes, means and standard deviations were extracted whenever reported. When continuous data were presented as medians with ranges or interquartile ranges, these were converted into estimated means and standard deviations using accepted statistical methods when appropriate [13]. When necessary, study authors were contacted for clarification of missing or unclear data. For trials reported across multiple publications (e.g., separate short-term and long-term reports), all available reports were used to extract data for the underlying randomized trial, ensuring that each patient cohort was counted only once in pooled analyses. When data were only available graphically, approximate values were extracted using digital tools when appropriate and clearly reported. All collected data were entered into a dedicated study database.

### Risk of bias and certainty assessment

The methodological quality of the included RCTs was independently assessed by two reviewers (FR, VL) using the Cochrane Risk of Bias 2 (RoB 2) tool [14]. The following domains were evaluated: bias from the randomization process, bias due to deviations from intended interventions, bias from missing outcome data, bias in outcome measurement, and bias in reporting results. Each domain was classified as low risk of bias, some concerns, or high risk of bias. Consistent with Cochrane methodology, risk-of-bias assessments were performed at the outcome level rather than solely at the study level: for each trial contributing to a pooled analysis, the applicability of each RoB 2 domain was evaluated separately for each synthesized outcome. Disagreements were resolved through consensus or arbitration by a third reviewer (CAS). Risk-of-bias plots were created using the robvis application (https://mcguinlu.shinyapps.io/robvis/). The certainty of evidence for each synthesized outcome was assessed using the GRADE approach and the GRADEpro GDT tool (Evidence Prime, Hamilton, Canada), with explicit domain-level justification for each downgrade [15].

### Data synthesis and statistical analysis

Qualitative and quantitative syntheses were conducted. A descriptive synthesis summarized study characteristics, patient populations, operative techniques, and outcomes that could not be pooled due to clinical or methodological heterogeneity. For dichotomous outcomes, pooled effect estimates were calculated as risk ratios (RR) with 95% confidence intervals using the Mantel–Haenszel method. For continuous outcomes, mean differences (MD) with 95% confidence intervals were calculated using the inverse-variance method when outcomes were measured on the same scale. A two-sided *p*-value below 0.05 was considered statistically significant. Software used for all statistical analyses was Review Manager (RevMan) version 5.4 (Cochrane Collaboration, Oxford, UK).

### Meta-analysis model and heterogeneity

Given the expected clinical variability across RCTs (such as differences in setting, surgeon experience, patient mix, and follow-up), pooled analyses primarily used a random-effects model. Statistical heterogeneity was evaluated with the *I*^2^ statistic and interpreted using standard thresholds (e.g., around 25% low, 50% moderate, 75% high) [16, 17], while also considering the direction and overlap of confidence intervals and the clinical comparability of the included studies.

## Results

### Study selection

**Fig. 1:**
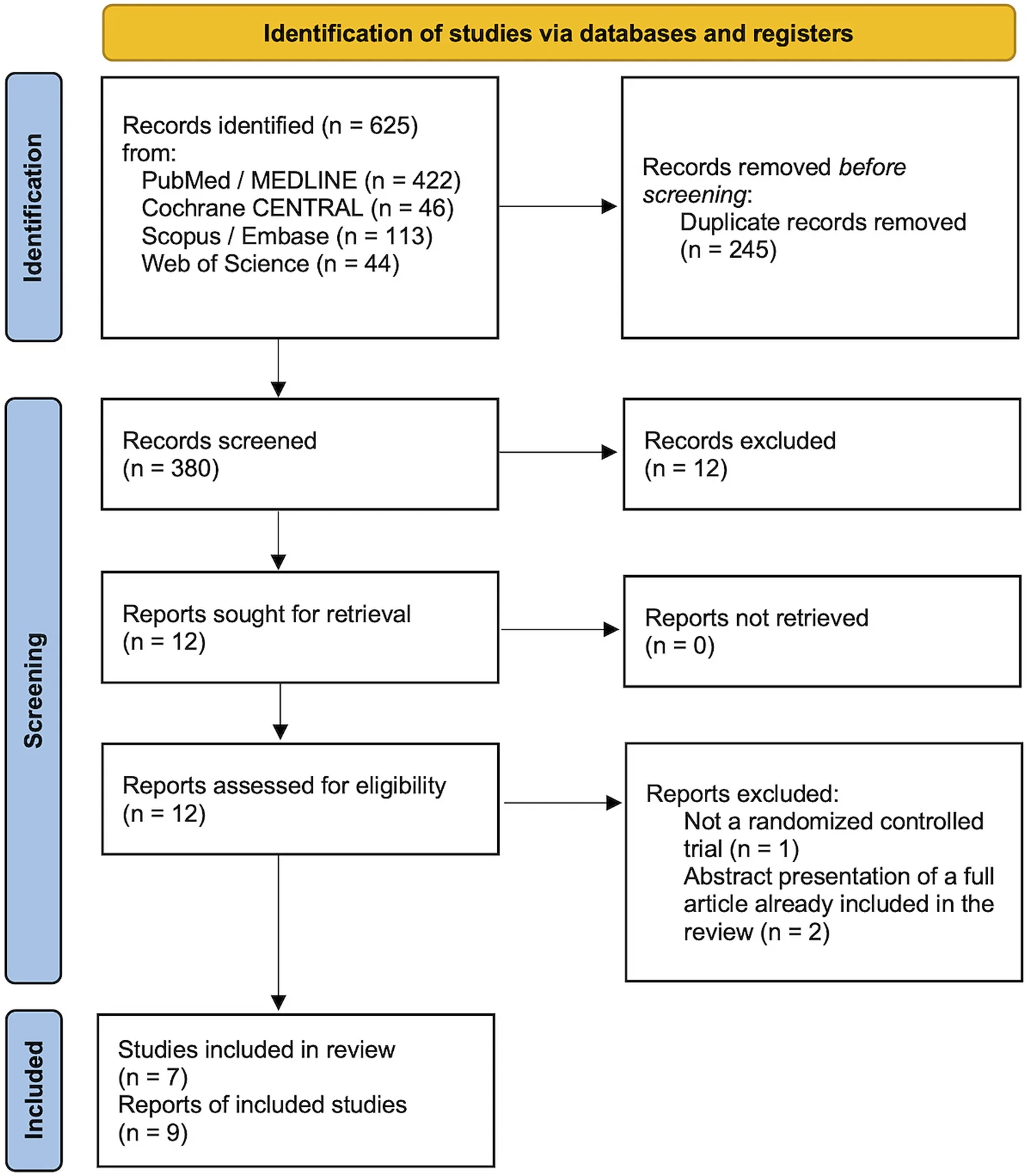
PRISMA 2020 flow diagram illustrating the study selection process. The final review included seven randomized controlled trials reported in nine articles; the discrepancy between trial and article counts reflects two pairs of publications sharing the same patient population: the RIVAL trial [18] and its long-term follow-up report (Miller et al. [19]), and the ROLAIS trial [24] and its operative time-profile analysis [22]

The database search yielded 625 records, of which 422 were identified through PubMed/MEDLINE, 46 through Cochrane CENTRAL, 113 through Scopus/Embase, and 44 through Web of Science. After removing duplicates, 380 records were screened by title and abstract. Twelve reports were considered potentially eligible and were reviewed in full text, with no retrieval failures. Three reports were excluded during the full-text review, including one non-randomized study and two abstract-only reports that corresponded to full-text articles already included in the review. In total, seven RCTs reported in nine articles were included in the final systematic review and meta-analysis [18–26]. A detailed overview of the selection process is provided in Fig. 1.

### Study characteristics

The studies were published between 2020 and 2026 and conducted across various healthcare settings, including centers in the USA, Switzerland, Denmark, and other countries, with most trials using a single-center design. Overall, the included trials assessed minimally invasive robotic versus laparoscopic inguinal hernia repair in adult patients with elective inguinal hernias, although the eligibility criteria and case mix varied across studies. The main design and clinical features of the seven included RCTs, reported in nine articles, are summarized in Table 1. Most RCTs compared r-TAPP to l-TAPP [18–24, 26], while the ROGER trial compared r-TAPP with l-TEP [25]. The populations included patients with unilateral and bilateral hernias, and some trials also included recurrent or inguinoscrotal hernias, whereas others focused solely on more selected elective cases. Baseline demographic and hernia characteristics were well balanced between treatment groups in most trials. The only studies showing significant baseline differences were the RIVAL trial (lower BMI in the robotic arm: 24.9 ± 3.24 vs. 26.9 ± 4.42 kg/m^2^; *p* = 0.014) [18], the VOLTAIRE trial (higher BMI in the robotic arm: 27.1 ± 3.6 vs. 24.2 ± 3.5 kg/m^2^; *p* = 0.004) [23], and the ROGER trial (greater proportion of lateral hernias [90% vs. 78%; *p* = 0.041] and larger defects exceeding 3 cm [56% vs. 39%; *p* = 0.026] in the r-TAPP group) [25]. A detailed overview of hernia laterality, hernia setting (primary vs. recurrent), and baseline comparability for each trial is provided in eTable 4. The VOLTAIRE trial utilized an open-console robotic platform (Versius; CMR Surgical, Cambridge, UK) [23], while all other trials used the da Vinci system (Intuitive Surgical, Sunnyvale, CA, USA) [18–22, 24–26]. Follow-up durations and focuses varied between studies. Some trials primarily assessed short-term postoperative outcomes, such as operative time, early pain, inflammatory markers, complications, same-day discharge, and surgeon workload or ergonomics, whereas others included medium- or long-term follow-up for outcomes like chronic pain, quality of life, and recurrence. Notably, the RIVAL trial provided both short-term randomized data [18] and longer-term 1- and 2-year follow-up results [19], which were considered multiple reports of the same underlying trial population.

**Table 1:** Characteristics of the included randomized controlled trials

Study (acronym)	Country	Setting	Comparison	Sample size (analyzed)	Hernia characteristics	Primary endpoint(s)	Follow-up
Prabhu et al. 2020 [18] (RIVAL)	USA	Multicenter	r-TAPP vs l-TAPP	102	Primary or recurrent Unilateral	Postoperative pain, QoL, mobility, wound morbidity, cosmesis	30 days
Miller et al. 2022 [19] (RIVAL follow-up)^†^	USA	Multicenter	r-TAPP vs l-TAPP	102 (48 rob, 54 lap)	Same as parent trial (above, Prabhu et al.)	Long-term pain, QoL, neuropathic pain, recurrence	1 and 2 years
Wikiel et al. 2023 [20]	USA	Single-center	r-TAPP vs l-TAPP	36 (19 rob, 17 lap)	Primary Unilateral	Stray energy thermal injury	36 months (mean)
Rodha et al. 2024 [21]^§^	India	Single-center	r-TAPP vs l-TAPP	101 (34 rob, 67 lap)	Unilateral or bilateral	Postoperative pain (VAS at 3, 6, 12 h)	1 and 4 weeks
Arunthavanathan et al. 2025 [22] (DIRECT)^‡^	Denmark	Single-center	r-TAPP vs l-TAPP	138 (74 rob, 64 lap)	Same as parent trial (below, Valorenzos et al.)	Operative time profile (4-step time analysis)	Short-term
Dixon et al. 2025 [23] (VOLTAIRE)^¶^	UK	Single-center	r-TAPP^¶^ vs l-TAPP	59 (39 rob, 20 lap)	Unilateral or bilateral inguinal hernia; elective; no inguinoscrotal or post-MIS recurrent	Surgeon ergonomic risk (REBA score)	14 days
Valorenzos et al. 2025 [24] (ROLAIS)	Denmark	Single-center	r-TAPP vs l-TAPP	139 (74 rob, 65 lap)	Primary or recurrent Unilateral or bilateral	Plasma CRP levels (POD 1 and 3)	6 months
Angehrn et al. 2026 [25] (ROGER)	Switzerland	Single-center	r-TAPP vs l-TEP	182 (91 per arm)	Primary Unilateral or bilateral	Postoperative pain while coughing at 24 h (NRS)	12 months
Chougala et al. 2026 [26]	India	Single-center	r-TAPP vs l-TAPP	72 (36 per arm)	Primary Unilateral or bilateral	Early postoperative pain (VAS at 8, 16, 24 h)	24 h

*CRP* C-reactive protein, *l-TAPP* laparoscopic transabdominal preperitoneal repair, *l-TEP* laparoscopic totally extraperitoneal repair, *MIS* minimally invasive surgery, *NRS* Numeric Rating Scale, *POD* postoperative day, *QoL* quality of life, *REBA* Rapid Entire Body Assessment, *r-TAPP* robotic transabdominal preperitoneal repair, *VAS* Visual Analogue Scale

^†^Miller et al. [19] represents a long-term follow-up report of the RIVAL randomized trial [18] and does not constitute an independent study population; it was included as a separate article to provide 1- and 2-year outcome data

^‡^Arunthavanathan et al. [22] represents a secondary analysis of the ROLAIS randomized trial [24], reporting a dedicated operative time-profile analysis from the same perspective randomized dataset. It does not constitute an independent study population

^§^Rodha et al. [21] is available as a conference abstract only and was not published as a full-text article at the time of this review

^¶^The VOLTAIRE trial [23] employed an open-console robotic platform (Versius; CMR Surgical, Cambridge, UK); all other trials used the da Vinci Xi system (Intuitive Surgical, Sunnyvale, CA, USA)

### Risk of bias

**Fig. 2:**
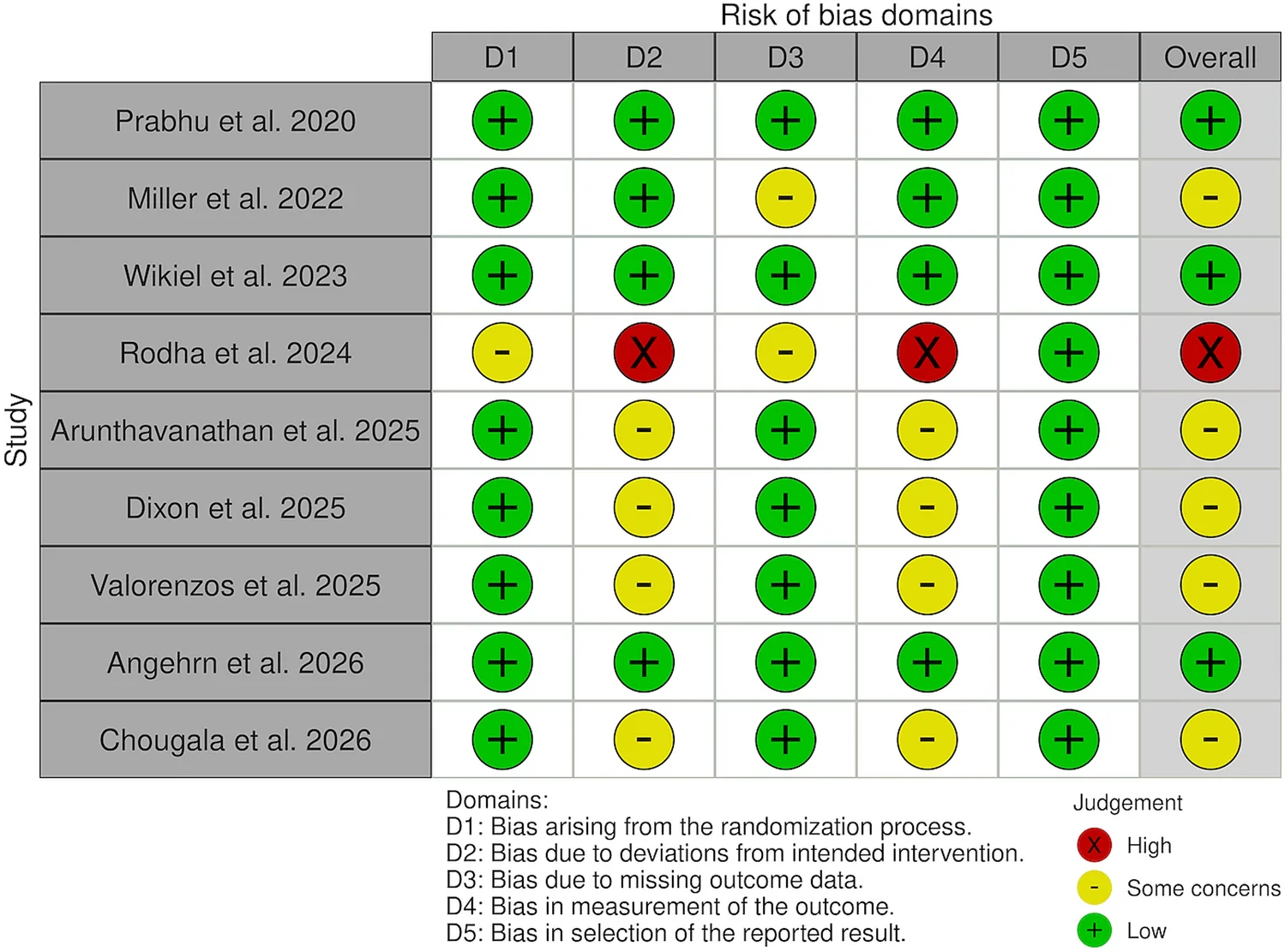
Risk of bias assessment of the included randomized controlled trials, generated using the robvis tool. Domain-level and overall judgments were assigned according to the Cochrane Risk of Bias 2 (RoB 2) tool across five domains: D1, bias arising from the randomization process; D2, bias due to deviations from intended interventions; D3, bias due to missing outcome data; D4, bias in measurement of outcomes; D5, bias in selection of reported results. Each domain was rated as low risk (green), some concerns (yellow), or high risk (red). The upper panel (traffic light plot) displays the domain-level judgments for each study; the lower panel (weighted bar chart) summarizes the distribution of judgments across all included studies. RIVAL [18] and Miller et al. [19] derive from the same patient population, as do the ROLAIS trial [24] and the DIRECT trial [22]. Study-level assessments shown here represent overall RoB 2 judgments; outcome-level assessments, which may differ for objective endpoints, are provided in eTable 5 (Color figure online)

Study-level overall assessments are summarized in Fig. 2 and detailed in eTable 5. Three RCTs were rated as having low overall risk of bias [18, 20, 25]. Five RCTs were identified as having some concerns [19, 22–24, 26]. Rodha et al.’s study was rated as having a high overall risk of bias [21] due to its abstract-only format, which prevented proper assessment of blinding, outcome measurement, and selective reporting.

Outcome-level assessments diverged from study-level judgments for objective outcomes: operative time and estimated blood loss were rated low for Domain 4 across all contributing trials, including those with open-label designs, given the instrumental nature of the measurements. Conversely, subjective endpoints, including pain, seroma, urinary retention, and discharge decisions, raised some concerns for Domain 4 in unblinded trials. Full outcome-level assessments are provided in eTable 5.

### Quantitative synthesis: primary outcomes

**Fig. 3:**
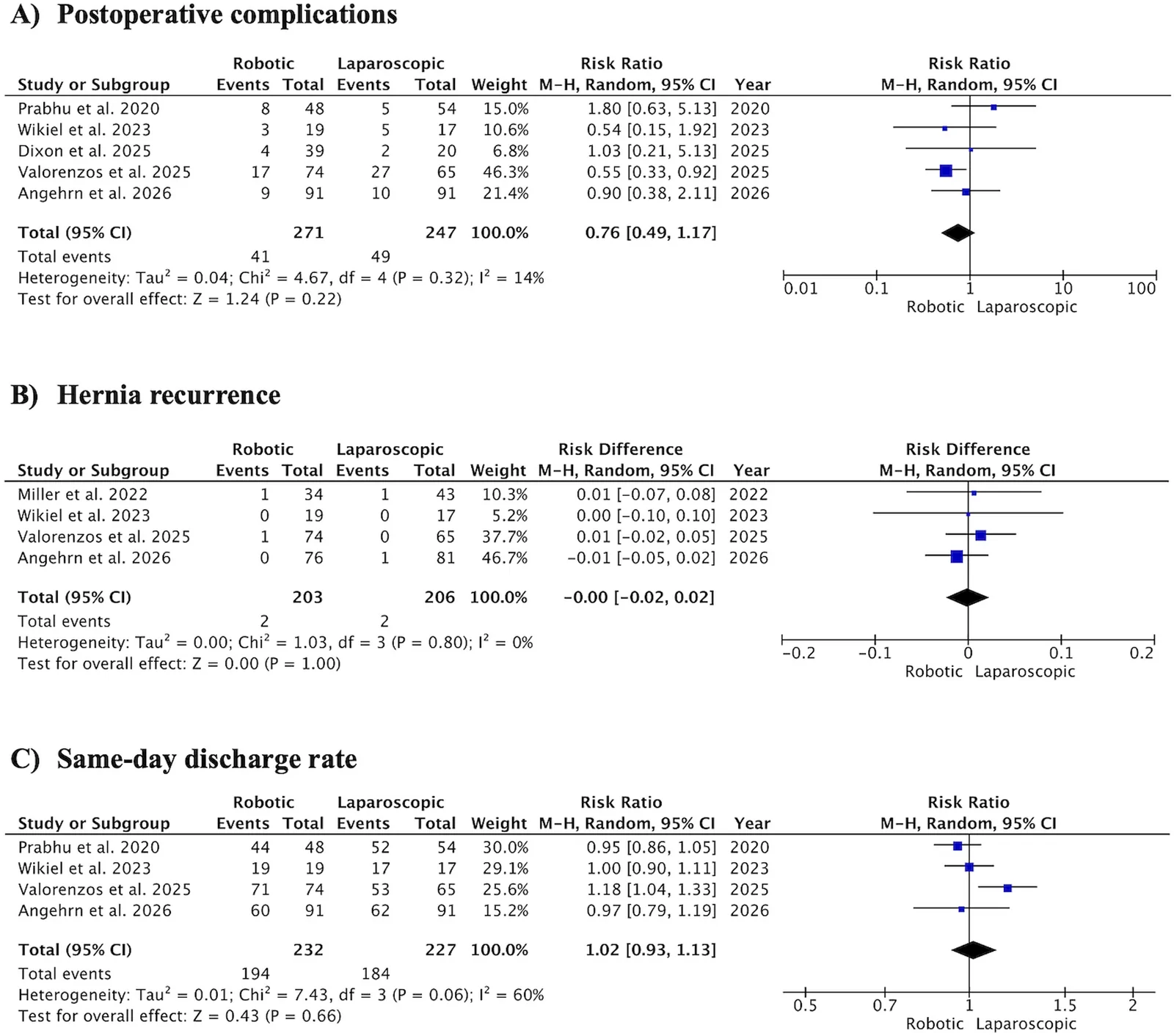
Forest plots for primary outcomes. **A** Overall postoperative complications, **B** hernia recurrence at longest follow-up, and **C** same-day discharge rate. Risk ratios with 95% confidence intervals or risk differences are shown for each trial and for the pooled estimate

*Overall postoperative complications* Five RCTs reporting data on 518 patients were included in the pooled analysis of overall postoperative complications [18, 20, 23–25]. Robotic repair was not associated with a significantly different complication rate compared with laparoscopic repair (RR 0.76, 95% CI 0.49–1.17; *I*^2^ = 14%; *p* = 0.22; Fig. 3A). The low level of heterogeneity indicates generally consistent findings across trials, although the confidence interval does not rule out a clinically meaningful reduction in favor of the robotic approach.

*Hernia recurrence* Recurrence data from the longest available follow-up were provided by 4 RCTs involving 409 patients [19, 20, 24, 25]. Overall event rates were very low, with only 2 recurrences in each group. Given the rarity of recurrence events (2 per group across 409 patients), a risk difference (RD) was calculated instead of an RR, consistent with Cochrane guidance for rare events. Pooled analysis showed no statistically significant difference between robotic and laparoscopic repair (RD − 0.00, 95% CI − 0.02 to 0.02; *I*^2^ = 0%; *p* = 1.00; Fig. 3B).

*Same-day discharge rate* Four RCTs, including 459 patients, reported same-day discharge rates [18, 20, 24, 25]. Robotic and laparoscopic repair achieved similar same-day discharge rates (RR 1.02, 95% CI 0.93–1.13; *I*^2^ = 60%; *p* = 0.66; Fig. 3C). Moderate heterogeneity was observed (*I*^2^ = 60%), likely reflecting differences in institutional protocols and discharge policies rather than true treatment effects.

*Acute postoperative pain* was evaluated across multiple trials using various measurement scales (VAS and NRS) and at inconsistent timepoints (ranging from 3–24 h to 7, 14, and 30 days after surgery), making meaningful quantitative analysis impossible. A narrative summary of the findings from individual trials on acute pain is included in the following descriptive section, along with other outcomes that cannot be pooled.

### Quantitative synthesis: secondary outcomes

The results of the pooled analyses for all pre-specified secondary outcomes are shown in eFig. 1. No statistically significant differences between groups were found for any endpoints. Operative time was analyzed in five studies (*n* = 518) [18, 20, 22, 23, 25] with subgroup analyses based on hernia laterality (Arunthavanathan et al. and Angehrn et al. provided separate data for unilateral and bilateral hernias). The overall pooled estimate was MD + 10.91 min (95% CI − 1.76 to 23.57; *I*^2^ = 98%; *p* = 0.09), with no significant difference in subgroups (*p* = 0.57) (see eFig. 1A). Estimated blood loss, analyzed in three RCTs (*n* = 234) [20, 23, 24], showed no significant difference between robotic and laparoscopic repair (MD − 0.29 ml, 95% CI − 1.44 to 0.86; *I*^2^ = 14%; *p* = 0.62; see eFig. 1B). Chronic postoperative pain was reported as a dichotomous outcome in two RCTs (*n* = 175) [20, 24] at 6–12 months (RR 0.53, 95% CI 0.20–1.39; *I*^2^ = 0%; *p* = 0.20; see eFig. 1C). Seroma (three RCTs, *n* = 277) [18, 20, 24], urinary retention (three RCTs, *n* = 277) [18, 20, 24], hematoma (two RCTs, *n* = 241) [18, 24], and hospital readmission (two RCTs, *n* = 198) [23, 24] showed no statistically significant differences between groups (RR 0.73 [0.17–3.25], RR 0.39 [0.09–1.71], RR 0.65 [0.10–4.45], and RR 0.31 [0.02–4.09], respectively; see eFigs. 1D–G).

### Qualitative synthesis: non-poolable outcomes

The abstract-only report by Rodha et al. was not included in any quantitative synthesis due to the lack of a full-text manuscript at the time of this review, which prevented a thorough assessment of methodology, risk of bias, and data completeness. Narratively, the trial reported no significant differences between robotic and laparoscopic TAPP in postoperative pain, overall operative time, length of hospital stay, urinary retention, or complication rates at 1- and 4-week follow-up, except for a shorter peritoneal dissection time in the robotic arm (*p* = 0.008) [21].

*Acute postoperative pain* was evaluated in six of the nine included studies. However, each trial used different measurement tools, scoring ranges, and postoperative timepoints, making quantitative comparisons impossible (see eTable 6). Five of the six trials found no statistically significant difference between groups: Prabhu et al. (VAS change from baseline at 1 week and 30 days; *p* = 0.86 and *p* = 0.85, respectively) [18], Wikiel et al. (binary presence vs. absence; *p* = 0.34) [20], Dixon et al. (NRS at day 14; *p* = 0.46) [23], Angehrn et al. (NRS at rest and during coughing at 24 h and 7 days; all *p* > 0.07) [25], and Rodha et al. (VAS at 3, 6, and 12 h; all *p* > 0.14) [21]. The only exception was Chougala et al., which reported significantly lower VAS scores in the robotic group at 8, 16, and 24 h postoperatively (overall mean VAS 3.38 ± 0.60 vs. 4.31 ± 0.50; *p* < 0.001) [26].

*Return to normal activity* Dixon et al. reported an unexpected finding regarding early functional recovery: a significantly higher proportion of patients in the robotic group had returned to normal daily activities at 14 days compared to the laparoscopic group (38.5% vs. 5%; *p* = 0.006), despite no significant difference in pain scores at the same timepoint [23].

*Surgical stress response* The ROLAIS trial specifically examined the surgical stress response as a primary or co-primary endpoint. Valorenzos et al. demonstrated that r-TAPP was associated with a significantly reduced systemic inflammatory response compared to l-TAPP, with CRP levels 23% lower on postoperative day 1 and 32% lower on postoperative day 3 in the robotic arm (*p* = 0.001) [24]. Plasma IL-6 levels were similarly decreased, showing a 26% reduction at 30 min post-extubation and 22% at 120 min in the robotic arm (*p* < 0.001) [24].

*Surgeon workload, ergonomics, and cognitive load* were evaluated across four trials using various instruments, resulting in differing outcomes. Prabhu et al. employed the Rapid Upper Limb Assessment (RULA) and found no significant difference in ergonomic burden between the robotic and laparoscopic arms (RULA grand score left: *p* = 0.31; right: *p* = 0.94). Additionally, NASA-TLX subscale scores for frustration (32.7 ± 23.5 vs. 20.1 ± 19.2; *p* = 0.004) and effort (36.7 ± 23.3 vs. 27.4 ± 24.1; *p* = 0.05) were higher in the robotic arm, indicating increased cognitive load with the da Vinci Xi system during the trial [18]. Conversely, Angehrn et al., also using the da Vinci Xi, observed significantly lower overall NASA-TLX workload scores for robotic surgery (18.4 ± 10.7 vs. 34.0 ± 17.2; *p* < 0.001), with only the mental demand subscale showing no significant difference [25]. Dixon et al., the only study using an open-console robotic platform (Versius; CMR Surgical), evaluated surgeon ergonomics via the Rapid Entire Body Assessment (REBA). The median REBA scores were notably lower for the robotic arm (median 3, IQR 2, classified as “low risk”) compared to the laparoscopic arm (median 5, IQR 1, classified as “medium risk”; *p* < 0.001). Surgeons spent 58% of operative time in a low-risk posture with the robotic approach versus 16% with laparoscopy. The total NASA-TLX score showed no significant difference between groups (19.9 ± 12.1 vs. 23.0 ± 11.2; *p* = 0.33), but the physical demand subscale was significantly lower for the robotic approach (2.8 ± 2.0 vs. 4.8 ± 1.8; *p* < 0.001) [23]. Chougala et al. evaluated surgeon fatigue using the Multidimensional Fatigue Symptom Inventory Short Form (MFSI-SF), reporting significantly better overall fatigue scores in the robotic arm (− 14.94 ± 2.98 vs. − 9.67 ± 2.65; *p* < 0.001 for most subscales) [26].

*Quality of life* Two trials reported formal assessments of quality of life. Prabhu et al. administered the SF-36 at baseline, 1 week, and 1 month, finding no significant differences between robotic and laparoscopic repair in any domain, including the Physical Component Summary and Mental Component Summary scores; both groups showed similar temporary declines at 1 week with full recovery by 1 month [18]. Angehrn et al. evaluated health utility at 12 months using the EQ-5D-5L and assessed inguinal pain perception at 24 h with the short-form Inguinal Pain Questionnaire (sf-IPQ), also reporting no significant differences between groups at any timepoint (EQ-5D-5L: *p* = 0.617; sf-IPQ: *p* = 0.840) [25].

*Procedural cost* Two RCTs reported data on procedural costs. Prabhu et al. found that robotic repair was linked to significantly higher total costs per case compared to laparoscopic repair (median $3258 [IQR $2568–$4118] vs. $1421 [IQR $1196–$1930]; *p* < 0.001) [18]. Similarly, Angehrn et al. reported higher procedural costs for robotic repair ($3327 ± 497 vs. $2732 ± 458; *p* < 0.001), although this analysis was limited to operative material costs for unilateral hernias and did not include capital equipment costs, maintenance, or hospital stay expenses [25].

*Stray energy and thermal injury* Wikiel et al. conducted the first in vivo RCT evaluating stray energy and thermal injury at trocar sites during TAPP repair [20]. Histological analysis of skin biopsies taken from port sites showed that thermal injury was detectable in 60% of samples from the robotic arm and 49% from the laparoscopic arm (25/51 samples), with no statistically significant difference (*p* = 0.548).

### Publication bias

Formal assessment of publication bias through funnel plot asymmetry was not conducted because the number of studies in each pooled analysis (ranging from 2 to 5) was too small to allow reliable visual or statistical evaluation. The minimum threshold of 10 studies per outcome was not reached for any endpoint. Planned sensitivity analyses could not be carried out due to the insufficient number of studies per outcome. Therefore, the possibility of publication bias cannot be ruled out and remains a limitation of this review.

### Certainty of evidence

The certainty of evidence for each outcome was evaluated using the GRADE approach with the GRADEpro GDT tool and is summarized in Table 2, with explicit domain-level justifications. The overall certainty was low to very low across all outcomes. For overall postoperative complications, hernia recurrence, and estimated blood loss, the certainty was rated low (⊕⊕○○), mainly due to risk of bias and imprecision. For all other outcomes, the certainty was rated very low (⊕○○○), mainly because of risk of bias, imprecision from sparse events and small sample sizes, and additional downgrading for inconsistency and indirectness (for operative time and hospital readmission).

**Table 2:** Certainty of evidence assessed using the GRADEpro GDT tool for outcomes included in the quantitative synthesis

Certainty assessment							№ of patients		Effect		Certainty	Importance
№ of studies	Study design	Risk of bias	Inconsistency	Indirectness	Imprecision	Other considerations	Robotic repair	Laparoscopic repair	Relative (95% CI)	Absolute(95% CI)		
Overall postoperative complications												
5	Randomized trials	Serious^a^	Not serious	Not serious	Serious^b^	None	41/271 (15.1%)	49/247 (19.8%)	RR 0.76 (0.49 to 1.17)	48 fewer per 1000 (from 101 fewer to 34 more)	⨁⨁◯◯ Low^a,b^	Critical
Hernia recurrence												
4	Randomized trials	Serious^c^	Not serious	Not serious	Serious^d^	None	2/203 (1.0%)	2/206 (1.0%)	RD 0.00 (− 0.02 to 0.02)	0 fewer per 1000 (from 12 fewer to 14 more)^e^	⨁⨁◯◯ Low^c,d^	Critical
Same-day discharge rate												
4	Randomized trials	Serious^f^	Serious^g^	Not serious	Serious^h^	None	194/232 (83.6%)	184/227 (81.1%)	RR 1.02 (0.93 to 1.13)	16 more per 1000 (from 57 fewer to 105 more)	⨁◯◯◯ Very low^f,g,h^	Critical
Operative time												
5	Randomized trials	Serious^i^	Very serious^j^	Serious^k^	Not serious	None	271	247	–	MD 10.91 min higher (1.76 lower to 23.57 higher)	⨁◯◯◯ Very low^i,j,k^	Important
Estimated blood loss												
3	Randomized trials	Not serious	Not serious	Not serious	Very serious^l^	None	132	102	–	MD 0.29 ml lower (1.44 lower to 0.86 higher)	⨁⨁◯◯ Low^l^	Important
Chronic postoperative pain												
2	Randomized trials	Serious^m^	Not serious	Not serious	Very serious^n^	None	6/93 (6.5%)	10/82 (12.2%)	RR 0.53 (0.20 to 1.39)	57 fewer per 1000 (from 98 fewer to 48 more)	⨁◯◯◯ Very low^m,n^	Critical
Seroma												
3	Randomized trials	Serious^o^	Serious^p^	Not serious	Very serious^q^	None	8/141 (5.7%)	9/136 (6.6%)	RR 0.73 (0.17 to 3.25)	18 fewer per 1000 (from 55 fewer to 149 more)	⨁◯◯◯ Very low^o,p,q^	Important
Urinary retention												
3	Randomized trials	Serious^r^	Not serious	Not serious	Very serious^s^	None	3/141 (2.1%)	9/136 (6.6%)	RR 0.39 (0.09 to 1.71)	40 fewer per 1000 (from 60 fewer to 47 more)	⨁◯◯◯ Very low^r,s^	Important
Hematoma												
2	Randomized trials	Serious^t^	Not serious	Not serious	Very serious^u^	None	6/122 (4.9%)	12/119 (10.1%)	RR 0.65 (0.10 to 4.45)	35 fewer per 1000 (from 91 fewer to 348 more)	⨁◯◯◯ Very low^t,u^	Important
Hospital readmission												
2	Randomized trials	Serious^v^	Not serious	Not serious	Very serious^w^	None	2/113 (1.8%)	8/85 (9.4%)	RR 0.31 (0.02 to 4.09)	65 fewer per 1000 (from 92 fewer to 291 more)	⨁◯◯◯ Very low^v,w^	Important

*CI* confidence interval, *MD* mean difference, *RR* risk ratio

^a^3/5 trials rated ‘some concerns’ on overall RoB 2 (Wikiel, Dixon, Valorenzos); outcome assessment unblinded in VOLTAIRE and ROLAIS (D4: some concerns); Angehrn low risk (patient-blinded)

^b^95%CI 0.52–1.09 spans from 48% RR to 9% RR

^c^Miller et al. rated ‘some concerns’ (D3: 19–25% loss to follow-up at 1–2 years); Valorenzos rated ‘some concerns’ (D2, D4); Angehrn low risk

^d^Only 2 events per arm (4 total)

^e^Given the rarity of recurrence events (2 per group across 409 patients), a risk difference (RD) was calculated instead of an RR, consistent with Cochrane guidance for rare events

^f^Discharge decisions unblinded in ROLAIS and VOLTAIRE; influenced by institutional protocols and surgeon preference (D4: some concerns); Prabhu and Angehrn low risk for this outcome

^g^*I*^2^ = 60%; Valorenzos RR 1.18 (1.04–1.33) vs Prabhu RR 0.95 (0.86–1.05) and Angehrn RR 0.97 (0.79–1.19); likely reflects institutional discharge policies rather than true treatment effect”

^h^95%CI 0.93–1.13; high baseline discharge rate (81%) creates ceiling effect

^i^Objective outcome but strongly influenced by surgeon experience and docking time; minimum robotic experience varies fivefold across trials (20 to ≥ 100 r-TAPPs)

^j^*I*^2^ = 98%; estimates range from MD − 20.0 min (Arunthavanathan, high-experience) to MD + 33.2 min (Dixon, minimum 20 procedures); opposite directions; divergence explained by surgeon experience tier, not chance

^k^ROGER (Angehrn) compares r-TAPP vs l-TEP; TEP involves different anatomical access, dissection planes, and docking requirements

^l^MD − 0.29 ml (95%CI − 1.44 to + 0.86); *n* = 234; floor effect likely (median near zero in all arms)

^m^Valorenzos rated ‘some concerns’ (D2, D4: unblinded subjective pain assessment at 6 months); Wikiel pain endpoint also unblinded

^n^2 trials, *n* = 175; 6 vs 10 events; RR 0.53 (95%CI 0.20–1.39)

^o^Seroma detection subjective and unblinded in RIVAL and ROLAIS (D4: some concerns); no standardized definition of clinically relevant seroma

^p^*I*^2^ = 47%; direction inconsistent: Prabhu favors laparoscopic; Wikiel and Valorenzos favor robotic

^q^8 vs 9 events; 95%CI 0.17–3.25 extremely wide

^r^Diagnosis unblinded in all three trials; catheterization protocols not standardized; anesthesia type (spinal vs general) not reported consistently (D4: some concerns in RIVAL and ROLAIS)

^s^3 vs 9 events; 95%CI 0.09–1.71

^t^Hematoma assessment unblinded in both trials; D4 rated some concerns in ROLAIS; no standardized imaging protocol for confirmation

^u^6 vs 12 events; 95%CI 0.10–4.45 extremely wide

^v^Both trials rated ‘some concerns’ on overall RoB 2 (VOLTAIRE: D2, D4; ROLAIS: D2, D4); readmission criteria not standardized across centers

^w^2 vs 8 events; 95%CI 0.02–4.09 extremely wide

## Discussion

This systematic review and meta-analysis compiles all RCT evidence comparing robotic and laparoscopic minimally invasive inguinal hernia repair, covering seven patient cohorts and 691 patients across an exceptionally broad range of clinical, patient-reported, and surgeon-centered endpoints. The main finding is clear: no statistically significant difference was found in any pre-specified pooled analysis, including overall postoperative complications, hernia recurrence, same-day discharge, or any secondary endpoint. This should not be interpreted as evidence of equivalence, which would require a formal non-inferiority trial design and sufficient statistical power, but rather as an honest acknowledgment of current evidence uncertainty, caused by an evidence base that remains underpowered for several outcomes. To understand why this uncertainty persists, despite nearly an 80-fold increase in robotic inguinal hernia repair adoption in the United States between 2008 and 2019 and a growing number of registered trials, it is important to carefully examine not only the results themselves but also the tools used to generate those results.

The most immediate barrier to evidence synthesis in this field is not statistical power but construct validity: among the six trials reporting acute postoperative pain, no two used the same measurement instrument, scoring range, or postoperative timepoint. Prabhu et al. used VAS change from baseline scores at 1 week and 30 days [18]; Wikiel et al. treated pain as a binary variable (presence vs. absence) [20]; Dixon et al. used the NRS at day 14 [23]; Angehrn et al. assessed NRS at rest and during coughing at 24 h and 7 days [25]; Chougala et al. measured VAS at 8, 16, and 24 h [26]; and Rodha et al. reported VAS at 3, 6, and 12 h [21]. This wide variation in measurement strategies is not a minor methodological detail but a fundamental obstacle to evidence synthesis. A VAS score at 8 h captures immediate anesthetic emergence pain, while a score at 30 days reflects the transition to subacute recovery, and a binary presence-versus-absence measure discards all information about severity. These are not simply different measures of the same construct; they represent different constructs. The fact that Chougala et al. is the only trial reporting significantly lower robotic arm pain, at timepoints (8–24 h) chosen by no other included trial, highlights how measurement timing can influence whether a statistically significant difference is found, regardless of any true treatment effect. The same issue applies to ergonomic and surgeon workload outcomes, where Prabhu et al. used RULA [18], Dixon et al. used REBA [23], Angehrn et al. used NASA-TLX alone [25], and Chougala et al. used the MFSI-SF fatigue inventory [26]: these instruments measure related but not identical constructs, preventing any quantitative pooling across trials.

These measurement issues highlight a more fundamental problem: statistical power. The fact that all pooled estimates were non-significant should be understood with epidemiological accuracy, not rhetorical ease. The 95% confidence interval for overall postoperative complications (RR 0.76, 95% CI 0.49–1.17) ranges from a 48% relative risk reduction to a 9% relative risk increase. The lower end of this interval indicates a clinically meaningful benefit that current data cannot rule out, while the upper end suggests a possible slight harm. The near-zero *I*^2^ (14%) for this endpoint indicates that the imprecision is not due to differences between trials but rather to low power: the trials tell a consistent story, but there are too few observations to resolve the uncertainty. Chronic postoperative inguinal pain is most affected by this lack of power. Data came from only two RCTs (175 patients), yielding a non-significant RR of 0.53 (95% CI 0.20–1. 39; *I*^2^ = 0%; *p* = 0.20): the point estimate suggests a 47% relative reduction that could be clinically important if true, but the lack of significance tells us nothing about whether a real effect exists. Recurrence faces a similar issue: four RCTs with 409 patients reported only two events per group, resulting in a pooled RD that is mathematically defined but not statistically meaningful. The same underpowering appears in the operative time analysis. Although a secondary endpoint, the extreme heterogeneity (*I*^2^ = 98%) is instructive: Prabhu et al. and Dixon et al. report robotic procedures taking 33–35 min longer [18, 23], whereas Arunthavanathan et al., the only phase-stratified analysis in the highest-experience group, reports the opposite, with significantly shorter robotic dissection times despite longer docking [22]. This divergence shows that operative time encompasses phases driven by fundamentally different platform-specific and experience-specific factors, and thus its pooled estimate is just the average of incompatible measures rather than a clear clinical conclusion.

Recognizing that the evidence base cannot yet fully answer the clinical question, it is important to examine why RCT results differ in direction across trials and identify which sources of between-trial variation can be addressed in future study designs. The most manageable of these is heterogeneity in surgeon experience, a confounding variable that cannot be eliminated by randomization because randomization addresses allocation bias, not proficiency differences. The minimum robotic experience required across included trials ranged from 20 procedures [23] to more than 100 certified r-TAPP procedures [24], a fivefold variation that reflects each trial’s position on the learning curve. Valorenzos et al., conducted exclusively by formally certified surgeons with ≥ 100 r-TAPPs, is the only trial showing a statistically significant reduction in complications [24]. Arunthavanathan et al., from the same population, is the only trial demonstrating shorter robotic dissection times [22]. In contrast, Prabhu et al. and Dixon et al., who require a minimum of 25 and 20 procedures, respectively, report longer robotic operative times and no advantage in complications [18, 23]. However, this pattern does not support the conclusion that robotic repair is superior in expert hands: the high-experience trials are underpowered to demonstrate superiority and include design features (such as more complex case mixes) that, independently, increase the observed complication rates in the laparoscopic group. Instead, this pattern indicates that experience level is a major factor in between-trial variation, and future trials should define surgeon eligibility based on a verified plateau point rather than a minimum case count, ideally using prospective CUSUM analysis as an eligibility criterion rather than a post hoc measure.

Given the widespread variability in measurements, limited statistical power, and confounding from learning curves, it is crucial to acknowledge the broader literature’s tendency to distort findings. A systematic review by Hansen et al. evaluated reporting practices across 35 studies comparing robotic with laparoscopic or open groin hernia repairs and found that spin-defined as reporting strategies that skew readers’ interpretation toward a more favorable view of an intervention-occurred in 57% of articles, with 100% prevalence among studies whose authors supported robotic surgery [27]. Affiliation with Intuitive Surgical was associated with a significantly higher rate of spin, with the most common form being the claim of equivalence based on non-significant results (40% of articles with spin), followed by exaggerated language and claims of benefit despite non-significant findings [27]. Notably, three RCTs in this review received funding from Intuitive Surgical [18, 19, 25], while the other trials were funded by independent academic or hospital sources or did not report their funding. Although industry funding does not necessarily compromise methodological quality, these three RCTs declared that the funder had no role in study design, analysis, or reporting. This funding pattern aligns with the documented association between higher spin prevalence and industry support, emphasizing the need for transparency when interpreting their results. This point is especially relevant to the current review: non-significant results from underpowered trials are necessary but not sufficient to declare equivalence, and the evidence should be communicated with this distinction explicitly maintained. The novel outcome measures explored, such as reduced surgical inflammatory response (CRP lowered by 23–32% and IL-6 by 26% in the robotic group) [24], no difference in stray thermal energy across platforms [20], and earlier return to normal activity despite similar pain scores [23], are genuine hypothesis-generating signals. However, their primary value lies in identifying questions for future adequately powered trials, rather than serving as evidence of superiority.

Regarding procedural costs, two RCTs reported consistent findings [18, 25]: robotic repair costs approximately USD 600–1800 more per case than laparoscopic repair when limited to operative material and personnel costs. These figures are probably underestimated, as neither analysis included capital acquisition costs, platform maintenance contracts, or instrument sterilization costs, and neither used formal cost-utility or cost-effectiveness methods. The main economic question for robotic inguinal hernia repair is whether any marginal clinical benefit from sufficiently powered future trials would outweigh the additional cost per quality-adjusted life-year, a question that current evidence cannot answer and that no included trial was designed to resolve.

This review has several limitations stemming from both the characteristics of the included evidence and the constraints inherent to any synthesis of a small body of literature. The maximum pooled sample size for any single outcome was 518 patients across five trials, which is insufficient to detect differences in rare outcomes with clinical precision, and the wide confidence intervals for all primary outcomes directly reflect this lack of power. Formal assessment of publication bias through funnel plots was not possible due to the small number of studies contributing data per outcome, so the possibility of selective reporting cannot be ruled out. The extreme statistical heterogeneity observed in operative time and the moderate heterogeneity in same-day discharge reflect genuine clinical and methodological diversity between trials that cannot be fully addressed through statistical modeling; therefore, pooled estimates for these endpoints should be interpreted with caution. Heterogeneity in outcome measurement was widespread, especially for acute pain, ergonomics, and surgeon workload, which prevented quantitative pooling for several pre-specified endpoints. Including the ROGER trial, which compares r-TAPP with l-TEP rather than l-TAPP as in all other studies, introduces a degree of clinical indirectness into the pooled analysis. This represents an inherent limitation of the comparative framework and is reflected in the GRADE downgrading for indirectness of outcomes influenced by this trial. Additionally, the lack of a predetermined number of studies per outcome (usually ≥ 7 for reliable meta-regression) prevented exploratory analyses of study-level covariates such as publication year, proportion of bilateral repairs, or surgeon experience, which could explain between-trial variability. Only three of seven included trials were rated as ‘low’ overall risk of bias using the RoB 2 tool, with five rated ‘some concerns’ and one rated ‘high’ risk. It should be noted that study-level and outcome-level RoB 2 assessments partially diverge: for objective outcomes measured independently of blinding status (operative time, blood loss), Domain 4 was rated low even in open-label trials, whereas subjective endpoints (pain, seroma, discharge) retained concerns for Domain 4 in unblinded settings. This distinction is reflected in the GRADE certainty ratings in Table 2 and detailed in eTable 5. The overall certainty of evidence, evaluated with the GRADE framework, was low to very low for all outcomes due to the combined effects of risk of bias, imprecision, and heterogeneity. This profile makes it methodologically impossible to translate these findings directly into practice recommendations without significant additional evidence. The review was limited to RCTs, which is appropriate for establishing causality but naturally restricts sample size and follow-up duration. The absence of data from lower-income healthcare settings further limits external validity, making the findings mainly applicable to high-volume, well-resourced robotic programs in high-income countries (a small segment of the global inguinal hernia population). Lastly, the broader evidence landscape contains structural gaps beyond this synthesis: variability across robotic platforms that precludes assuming uniform effects; a lack of embedded health-economic analyses using formal cost-utility methods; no standardized core outcome sets for minimally invasive hernia repair; predominantly single-center trials with few surgeons, which limits external validity; and underrepresentation of complex hernias (bilateral, recurrent, inguinoscrotal) and high-BMI cases, possibly the groups most likely to benefit from platform-specific advantages.

In conclusion, across seven randomized controlled trial cohorts, robotic minimally invasive inguinal hernia repair showed no statistically significant difference from laparoscopic repair in any pre-specified outcome. The certainty of evidence was low for overall postoperative complications, hernia recurrence, and estimated blood loss, and very low for all other outcomes, mainly due to small sample sizes, heterogeneity across outcomes, and bias in subjective outcome measurement. These findings reflect an evidence base that does not yet permit reliable conclusions about comparative effectiveness between techniques.

## Supplementary Information

Below is the link to the electronic supplementary material.

Supplementary file1 (PDF 1022 KB)

## Data Availability

All the data are available upon reasonable request.
