# Supplementary material for: Robotic versus laparoscopic inguinal hernia repair: a systematic review and meta-analysis of randomized controlled trials
Source: Surg Endosc. 2026 Aug 20;40(9):7411–25. doi: 10.1007/s00464-026-13253-y (PMC13582080; doi:10.1007/s00464-026-13253-y)

Supplemental Material

Manuscript title: "Robotic Versus Laparoscopic Inguinal Hernia Repair: Non-Significance Is Not Equivalence. *A Systematic Review and Meta-Analysis of Randomized Controlled Trials*"

eTable 1. PRISMA 2020 checklist
eTable 2. Search equations
eTable 3. Summary of the studies included and excluded on the full-text analysis
eTable 4. Surgical technique, hernia laterality, hernia setting (primary vs. recurrent), and significant baseline demographic differences by study.
eTable 5. Risk-of-bias assessments at the outcome level: domain-specific judgements for each trial–outcome combination.
eTable 6. Structured overview of acute postoperative pain assessment across included trials.
eFigure 1. Forrest plots – Secondary outcomes

eTable 1. PRISMA 2020 checklist

Section and Topic	Item #	Checklist item	Location where item is reported
**TITLE**			
Title	1	Identify the report as a systematic review.	Title page
**ABSTRACT**			
Abstract	2	See the PRISMA 2020 for Abstracts checklist.	Abstract
**INTRODUCTION**			
Rationale	3	Describe the rationale for the review in the context of existing knowledge.	Introduction, paragraphs 1–3
Objectives	4	Provide an explicit statement of the objective(s) or question(s) the review addresses.	Introduction, final paragraph
**METHODS**			
Eligibility criteria	5	Specify the inclusion and exclusion criteria for the review and how studies were grouped for the syntheses.	Methods — Eligibility criteria
Information sources	6	Specify all databases, registers, websites, organisations, reference lists and other sources searched or consulted to identify studies. Specify the date when each source was last searched or consulted.	Methods — Literature search strategy
Search strategy	7	Present the full search strategies for all databases, registers and websites, including any filters and limits used.	Methods — Literature search strategy; eTable 2
Selection process	8	Specify the methods used to decide whether a study met the inclusion criteria of the review, including how many reviewers screened each record and each report retrieved, whether they worked independently, and if applicable, details of automation tools used in the process.	Methods — Study selection
Data collection process	9	Specify the methods used to collect data from reports, including how many reviewers collected data from each report, whether they worked independently, any processes for obtaining or confirming data from study investigators, and if applicable, details of automation tools used in the process.	Methods — Data extraction
Data items	10a	List and define all outcomes for which data were sought. Specify whether all results that were compatible with each outcome domain in each study were sought (e.g. for all measures, time points, analyses), and if not, the methods used to decide which results to collect.	Methods — Eligibility criteria (Outcomes); Data extraction
	10b	List and define all other variables for which data were sought (e.g. participant and intervention characteristics, funding sources). Describe any assumptions made about any missing or unclear information.	Methods — Data extraction
Study risk of bias assessment	11	Specify the methods used to assess risk of bias in the included studies, including details of the tool(s) used, how many reviewers assessed each study and whether they worked independently, and if applicable, details of automation tools used in the process.	Methods — Risk of bias and certainty assessment
Effect measures	12	Specify for each outcome the effect measure(s) (e.g. risk ratio, mean difference) used in the synthesis or presentation of results.	Methods — Data synthesis and statistical analysis
Synthesis methods	13a	Describe the processes used to decide which studies were eligible for each synthesis (e.g. tabulating the study intervention characteristics and comparing against the planned groups for each synthesis (item #5)).	Methods — Data synthesis
	13b	Describe any methods required to prepare the data for presentation or synthesis, such as handling of missing summary statistics, or data conversions.	Methods — Data synthesis
	13c	Describe any methods used to tabulate or visually display results of individual studies and syntheses.	Results — forest plots (Figure 3; eFigure 1)
	13d	Describe any methods used to synthesize results and provide a rationale for the choice(s). If meta-analysis was performed, describe the model(s), method(s) to identify the presence and extent of statistical heterogeneity, and software package(s) used.	Methods — Meta-analysis model and heterogeneity
	13e	Describe any methods used to explore possible causes of heterogeneity among study results (e.g. subgroup analysis, meta-regression).	Methods — Meta-analysis model; Results (subgroup operative time)
	13f	Describe any sensitivity analyses conducted to assess robustness of the synthesized results.	Methods — Data synthesis (not performed — insufficient studies)

Section and Topic	Item #	Checklist item	Location where item is reported
Reporting bias assessment	14	Describe any methods used to assess risk of bias due to missing results in a synthesis (arising from reporting biases).	Methods — Publication bias; Results — Publication bias
Certainty assessment	15	Describe any methods used to assess certainty (or confidence) in the body of evidence for an outcome.	Methods — Risk of bias and certainty assessment
**RESULTS**			
Study selection	16a	Describe the results of the search and selection process, from the number of records identified in the search to the number of studies included in the review, ideally using a flow diagram.	Results — Study selection; Figure 1
	16b	Cite studies that might appear to meet the inclusion criteria, but which were excluded, and explain why they were excluded.	Supplementary Material — eTable 3
Study characteristics	17	Cite each included study and present its characteristics.	Results — Study characteristics; Table 1
Risk of bias in studies	18	Present assessments of risk of bias for each included study.	Results — Risk of bias; Figure 2; Supplementary Material — eTable 5
Results of individual studies	19	For all outcomes, present, for each study: (a) summary statistics for each group (where appropriate) and (b) an effect estimate and its precision (e.g. confidence/credible interval), ideally using structured tables or plots.	Results — Quantitative synthesis; Table 1; Figure 3; eFigure 1
Results of syntheses	20a	For each synthesis, briefly summarise the characteristics and risk of bias among contributing studies.	Results — Quantitative synthesis
	20b	Present results of all statistical syntheses conducted. If meta-analysis was done, present for each the summary estimate and its precision (e.g. confidence/credible interval) and measures of statistical heterogeneity. If comparing groups, describe the direction of the effect.	Results — Quantitative synthesis; Figure 3; eFigure 1
	20c	Present results of all investigations of possible causes of heterogeneity among study results.	Results — Quantitative synthesis (subgroup analyses)
	20d	Present results of all sensitivity analyses conducted to assess the robustness of the synthesized results.	Results — Publication bias (not performed)
Reporting biases	21	Present assessments of risk of bias due to missing results (arising from reporting biases) for each synthesis assessed.	Results — Publication bias
Certainty of evidence	22	Present assessments of certainty (or confidence) in the body of evidence for each outcome assessed.	Results — Certainty of evidence; Table 2
**DISCUSSION**			
Discussion	23a	Provide a general interpretation of the results in the context of other evidence.	Discussion, paragraphs 1–6
	23b	Discuss any limitations of the evidence included in the review.	Discussion — Limitations
	23c	Discuss any limitations of the review processes used.	Discussion — Limitations
	23d	Discuss implications of the results for practice, policy, and future research.	Discussion — Limitations; Conclusions
**OTHER INFORMATION**			
Registration and protocol	24a	Provide registration information for the review, including register name and registration number, or state that the review was not registered.	Methods — Study design; PROSPERO CRD420261370145
	24b	Indicate where the review protocol can be accessed, or state that a protocol was not prepared.	Methods — Study design (PROSPERO)
	24c	Describe and explain any amendments to information provided at registration or in the protocol.	Not applicable
Support	25	Describe sources of financial or non-financial support for the review, and the role of the funders or sponsors in the review.	Funding statement
Competing interests	26	Declare any competing interests of review authors.	Conflict of interest statement
Availability of data, code and other materials	27	Report which of the following are publicly available and where they can be found: template data collection forms; data extracted from included studies; data used for all analyses; analytic code; any other materials used in the review.	Data availability statement

eTable 2. Search equations

Database	Search equation	Results
**PubMed / MEDLINE**	("Hernia, Inguinal"[Mesh] OR inguinal hernia*[tiab] OR groin hernia*[tiab]) AND (robotic[tiab] OR robot-assisted[tiab] OR robot assisted[tiab] OR robot*[tiab]) AND (laparoscopic[tiab] OR laparoscopy[Mesh])	422
**Cochrane CENTRAL**	(inguinal hernia OR groin hernia) AND (robotic OR robot-assisted OR robot*) AND (laparoscopic OR minimally invasive)	46
**Scopus / Embase**	TITLE-ABS-KEY(("inguinal hernia" OR "groin hernia") AND (robotic OR "robot assisted" OR robot*) AND (laparoscopic OR "minimally invasive")	113
**Web of Science**	TS=(("inguinal hernia" OR "groin hernia") AND (robotic OR "robot assisted" OR robot*) AND (laparoscopic OR "minimally invasive")	44
		**Total = 625**

Searches results downloaded on: 13 April 2026

eTable 3. Summary of the studies included and excluded on the full-text analysis

Author (year)	Title	Inclusion?	Reason for exclusion
Aghayeva (2020)	Laparoscopic totally extraperitoneal vs robotic transabdominal preperitoneal inguinal hernia repair: Assessment of short- and long-term outcomes	Excluded	Not a randomized controlled trial
Angehrn (2025)	Robotic Versus Conventional Minimally Invasive Inguinal Hernia Repair: The Blinded Randomized Controlled ROGER Trial	Excluded	Abstract presentation of a full article already included in the review (see below)
Angehrn (2026)	Robotic versus laparoscopic minimally invasive inguinal hernia repair: randomized clinical trial (the ROGER trial)	Included	
Arunthavanathan (2025)	Shorter operative times following robotic-assisted transabdominal preperitoneal inguinal hernia repair (TAPP) compared to laparoscopic TAPP: the Danish Inguinal Randomized Controlled Trial (DIRECT)	Included	
Chougala (2026)	Robotic-assisted versus laparoscopic transabdominal preperitoneal inguinal hernia repair: A cross-sectional comparison of surgical outcomes	Included	
Dixon (2025)	Robotic assisted surgery reduces ergonomic risk during minimally invasive inguinal hernia repair: the VOLTAIRE randomised controlled trial	Included	
Miller (2022)	Long-term outcomes of the RIVAL trial: 1- and 2-year outcomes of a prospective, randomized clinical trial comparing laparoscopic versus robotic inguinal hernia repair	Excluded	Abstract presentation of a full article already included in the review (see below)
Miller (2022)	Laparoscopic versus robotic inguinal hernia repair: 1- and 2-year outcomes from the RIVAL trial	Included	
Prabhu (2020)	Robotic Inguinal vs Transabdominal Laparoscopic Inguinal Hernia Repair The RIVAL Randomized Clinical Trial	Included	
Rodha (2024)	Laparoscopic Versus Robotic Trans Abdominal Preperitoneal Inguinal Hernia Repair: A Randomized Controlled Trial	Included	
Valorenzos (2025)	Inflammatory response and short-term outcomes after laparoscopic versus robotic transabdominal preperitoneal inguinal hernia repair: randomized clinical trial (ROLAIS)	Included	
Wikiel (2023)	Stray energy injury during robotic versus laparoscopic inguinal hernia repair: a randomized controlled trial	Included	

eTable 4. Surgical technique, hernia laterality, hernia setting (primary vs. recurrent), and significant baseline demographic differences by study.

Study (acronym)	Comparison	Unilateral, n (%)	Bilateral, n (%)	Primary, n (%)	Recurrent, n (%)	Significant baseline differences between groups
Prabhu et al. 2020 (RIVAL)	r-TAPP vs l-TAPP	48 (100) vs 54 (100)	0 (0) vs 0 (0)	43 (89.4) vs 51 (94.4)	5 (10.6) vs 3 (5.6)	BMI: Rob 24.9±3.24 vs Lap 26.9±4.42, P=0.014
Miller et al. 2022 (RIVAL follow-up) †	r-TAPP vs l-TAPP	Same cohort as parent trial above (Prabhu et al.)				
Wikiel et al. 2023	r-TAPP vs l-TAPP	19 (100) vs 17 (100)	0 (0) vs 0 (0)	19 (100) vs 17 (100)	0 (0) vs 0 (0)	None
Rodha et al. 2024 §	r r-TAPP vs l-TAPP	27 (79.4) vs 58 (86.6)	7 (20.6) vs 9 (13.4)	NR	NR	None
Arunthavanathan et al. 2025 (DIRECT) ‡	r-TAPP vs l-TAPP	Same cohort as parent trial below (Valorenzos et al.)				
Dixon et al. 2025 (VOLTAIRE) ¶	r-TAPP vs l-TAPP	36 (92.3) vs 18 (90)	3 (7.7) vs 2 (10)	39 (100) vs 20 (100)	0 (0) vs 0 (0)	BMI: Rob 27.1±3.6 vs Lap 24.2±3.5, P=0.004
Valorenzos et al. 2025 (ROLAIS)	r-TAPP vs l-TAPP	58 (78.4) vs 57 (87.7)	16 (21.6) vs 8 (12.3)	67 (90.5) vs 58 (89.2)	7 (9.5) vs 7 (10.8)	None
Angehrn et al. 2026 (ROGER)	r-TAPP vs l-TEP	72 (79.1) vs 71 (78)	19 (20.9) vs 20 (22)	91 (100) vs 91 (100)	0 (0) vs 0 (0)	Hernia type distribution: more lateral hernias (90% vs 78%, P=0.041) and type III (>3 cm) (56% vs 39%, P=0.026) in rTAPP vs TEP
Chougala et al. 2026	r-TAPP vs l-TAPP	13 (36.1) vs 13 (36.1)	23 (63.9) vs 23 (63.9)	36 (100) vs 36 (100)	0 (0) vs 0 (0)	None

Abbreviations: BMI, body mass index; l-TAPP, laparoscopic transabdominal preperitoneal repair; l-TEP, laparoscopic totally extraperitoneal repair; NR, not reported; r-TAPP, robotic transabdominal preperitoneal repair.

Notes:

†Miller et al. (2022) represents a long-term follow-up report of the RIVAL randomized trial (Prabhu et al., 2020) and does not constitute an independent study population; it was included as a separate article to provide 1- and 2-year outcome data.

‡Arunthavanathan et al. (2025) represents a secondary analysis of the ROLAIS randomized trial (Valorenzos et al., 2025), reporting a dedicated operative time-profile analysis from the same perspective randomized dataset. It does not constitute an independent study population.

§Rodha et al. (2024) is available as a conference abstract only and was not published as a full-text article at the time of this review.

¶The VOLTAIRE trial (Dixon et al., 2025) employed an open-console robotic platform (Versius; CMR Surgical, Cambridge, UK); all other trials used the da Vinci Xi system (Intuitive Surgical, Sunnyvale, CA, USA).

eTable 5. Risk-of-bias assessments at the outcome level: domain-specific judgements for each trial–outcome combination.

1. Overall postoperative complications

Study	D1	D2	D3	D4	D5	Outcome-level overall
Prabhu et al. 2020 (RIVAL)	+	+	+	+	+	Low
Wikiel et al. 2023	+	+	+	+	+	Low
Dixon et al. 2025 (VOLTAIRE)	+	−	+	−	+	Some concerns
Valorenzos et al. 2025 (ROLAIS)	+	−	+	−	+	Some concerns
Angehrn et al. 2026 (ROGER)	+	+	+	+	+	Low

2. Hernia recurrence

Study	D1	D2	D3	D4	D5	Outcome-level overall
Miller et al. 2022 (RIVAL follow-up)	+	+	−	+	+	Some concerns
Wikiel et al. 2023	+	+	+	+	+	Low
Valorenzos et al. 2025 (ROLAIS)	+	−	+	−	+	Some concerns
Angehrn et al. 2026 (ROGER)	+	+	+	+	+	Low

3. Same-day discharge rate

Study	D1	D2	D3	D4	D5	Outcome-level overall
Prabhu et al. 2020 (RIVAL)	+	+	+	+	+	Low
Wikiel et al. 2023	+	+	+	+	+	Low
Valorenzos et al. 2025 (ROLAIS)	+	−	+	−	+	Some concerns
Angehrn et al. 2026 (ROGER)	+	+	+	+	+	Low

4. Operative time

Study	D1	D2	D3	D4	D5	Outcome-level overall
Prabhu et al. 2020 (RIVAL)	+	+	+	+	+	Low
Arunthavanathan et al. 2025 (DIRECT)	+	−	+	+	+	Some concerns
Dixon et al. 2025 (VOLTAIRE)	+	−	+	+	+	Some concerns
Valorenzos et al. 2025 (ROLAIS)	+	−	+	+	+	Some concerns
Angehrn et al. 2026 (ROGER)	+	+	+	+	+	Low

5. Estimated blood loss

Study	D1	D2	D3	D4	D5	Outcome-level overall
Wikiel et al. 2023	+	+	+	+	+	Low

Study	D1	D2	D3	D4	D5	Outcome-level overall
Valorenzos et al. 2025 (ROLAIS)	+	−	+	+	+	Some concerns
Dixon et al. 2025 (VOLTAIRE)	+	−	+	+	+	Some concerns

6. Chronic postoperative pain

Study	D1	D2	D3	D4	D5	Outcome-level overall
Wikiel et al. 2023	+	+	+	−	+	Some concerns
Valorenzos et al. 2025 (ROLAIS)	+	−	+	−	+	Some concerns

7. Seroma

Study	D1	D2	D3	D4	D5	Outcome-level overall
Prabhu et al. 2020 (RIVAL)	+	+	+	+	+	Low
Wikiel et al. 2023	+	+	+	+	+	Low
Valorenzos et al. 2025 (ROLAIS)	+	−	+	−	+	Some concerns

8. Urinary retention

Study	D1	D2	D3	D4	D5	Outcome-level overall
Prabhu et al. 2020 (RIVAL)	+	+	+	+	+	Low
Wikiel et al. 2023	+	+	+	+	+	Low
Valorenzos et al. 2025 (ROLAIS)	+	−	+	−	+	Some concerns

9. Hematoma

Study	D1	D2	D3	D4	D5	Outcome-level overall
Prabhu et al. 2020 (RIVAL)	+	+	+	+	+	Low
Valorenzos et al. 2025 (ROLAIS)	+	−	+	−	+	Some concerns

10. Hospital readmission

Study	D1	D2	D3	D4	D5	Outcome-level overall
Dixon et al. 2025 (VOLTAIRE)	+	−	+	−	+	Some concerns
Valorenzos et al. 2025 (ROLAIS)	+	−	+	−	+	Some concerns

Abbreviations: D1–D5, RoB 2 domains 1–5; l-TAPP, laparoscopic transabdominal preperitoneal repair; l-TEP, laparoscopic totally extraperitoneal repair; r-TAPP, robotic transabdominal preperitoneal repair; VAS, visual analogue scale.

Note: Outcome-level assessments may differ from study-level overall judgments when the nature of the specific outcome (objective vs. subjective) modifies the applicability of blinding-related domains (D2, D4). In particular, objective outcomes recorded independently of observer awareness (operative time, blood loss, histopathology) are not downgraded for D4 in open-label trials, consistent with the Cochrane RoB 2 guidance on the application of the outcome-specific domain.

eTable 6. Structured overview of acute postoperative pain assessment across included trials

Study	Measurement instrument	Scale range	Timepoint(s)	P-value	Significant difference
Prabhu et al. 2020 (RIVAL)	VAS (change from baseline)	0–100 mm	1 week; 30 days	p=0.86 (1 wk); p=0.85 (30 d)	No
Wikiel et al. 2023	Binary (presence vs. absence)	Yes/No	Not specified	p=0.34	No
Rodha et al. 2024 §	VAS	0–10	3 h; 6 h; 12 h	All p>0.14	No
Dixon et al. 2025 (VOLTAIRE)	NRS	0–10	Day 14	p=0.46	No
Angehrn et al. 2026 (ROGER)	NRS (at rest and during coughing)	0–10	24 h; 7 days; 30 days	p=0.431; p=0.964; p=0.074	No
Chougala et al. 2026	VAS	0–10	8 h; 16 h; 24 h	p<0.001	Yes

Abbreviations: h, hours; IQR, interquartile range; NRS, Numeric Rating Scale; VAS, Visual Analogue Scale; wk, week.
§ Rodha et al. (2024) is available as a conference abstract only; individual arm data were not reported.

eFigure 1. Forrest plots – Secondary outcomes

A) Operative time

Study or Subgroup	Robotic Mean	Robotic SD	Robotic Total	Laparoscopic Mean	Laparoscopic SD	Laparoscopic Total	Weight	Mean Difference IV, Random, 95% CI	Year
**1.10.1 Unilateral**									
Prabhu et al. 2020	76.1	25.7	48	44.5	25.6	54	14.1%	31.60 [21.63, 41.57]	2020
Wikiel et al. 2023	86.5	23	19	82.1	22.7	17	12.7%	4.40 [−10.55, 19.35]	2023
Arunthavanathan et al. 2025 (1)	51	3	43	60	3	38	15.4%	−9.00 [−10.31, −7.69]	2025
Dixon et al. 2025	64.9	21.1	39	31.7	10.3	20	14.5%	33.20 [25.19, 41.21]	2025
Anghern et al. 2026 (1)	80.3	20.9	72	64.2	19.2	71	14.8%	16.10 [9.52, 22.68]	2026
**Subtotal (95% CI)**			**221**			**200**	**71.6%**	**15.21 [−5.52, 35.94]**	
Heterogeneity: Tau² = 537.46; Chi² = 211.66, df = 4 (P < 0.00001); I² = 98%									
Test for overall effect: Z = 1.44 (P = 0.15)									
**1.10.2 Bilateral**									
Arunthavanathan et al. 2025 (2)	72	4	31	92	7	27	15.3%	−20.00 [−22.99, −17.01]	2025
Anghern et al. 2026 (2)	126.6	19.8	19	102.7	23.8	20	13.1%	23.90 [10.19, 37.61]	2026
**Subtotal (95% CI)**			**50**			**47**	**28.4%**	**1.42 [−41.59, 44.43]**	
Heterogeneity: Tau² = 937.96; Chi² = 37.58, df = 1 (P < 0.00001); I² = 97%									
Test for overall effect: Z = 0.06 (P = 0.95)									
**Total (95% CI)**			**271**			**247**	**100.0%**	**10.91 [−1.76, 23.57]**	
Heterogeneity: Tau² = 270.25; Chi² = 301.06, df = 6 (P < 0.00001); I² = 98%									
Test for overall effect: Z = 1.69 (P = 0.09)									
Test for subgroup differences: Chi² = 0.32, df = 1 (P = 0.57), I² = 0%									

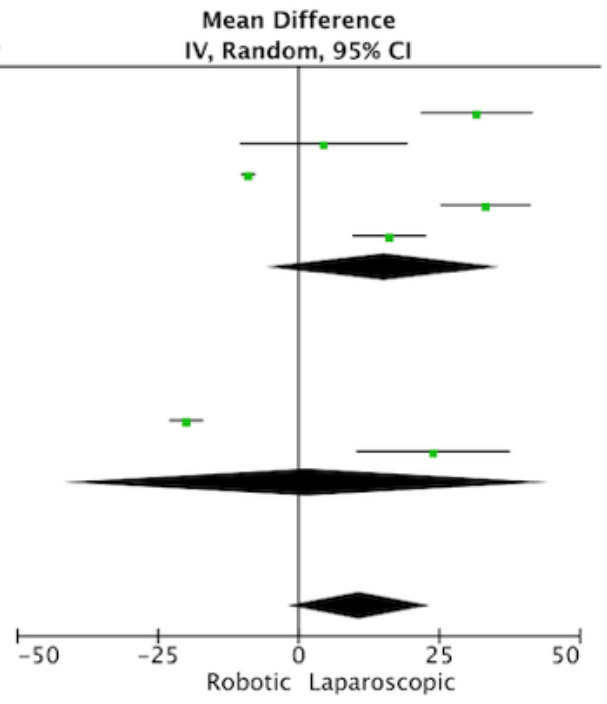

B) Estimated blood loss

Study or Subgroup	Robotic Mean	Robotic SD	Robotic Total	Laparoscopic Mean	Laparoscopic SD	Laparoscopic Total	Weight	Mean Difference IV, Random, 95% CI	Year
Wikiel et al. 2023	14.5	10.1	19	14.6	9.7	17	3.1%	−0.10 [−6.57, 6.37]	2023
Valorenzos et al. 2025	5	7.4	74	7.5	11.1	65	11.5%	−2.50 [−5.68, 0.68]	2025
Dixon et al. 2025	10	0.7	39	10	0.7	20	85.5%	0.00 [−0.38, 0.38]	2025
**Total (95% CI)**			**132**			**102**	**100.0%**	**−0.29 [−1.44, 0.86]**	
Heterogeneity: Tau² = 0.36; Chi² = 2.34, df = 2 (P = 0.31); I² = 14%									
Test for overall effect: Z = 0.49 (P = 0.62)									

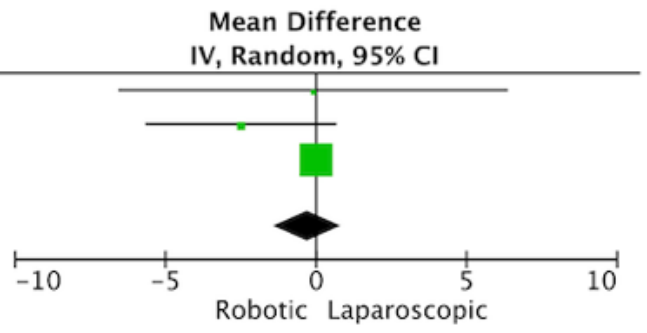

C) Chronic pain

Study or Subgroup	Robotic Events	Robotic Total	Laparoscopic Events	Laparoscopic Total	Weight	Risk Ratio M-H, Random, 95% CI	Year
Wikiel et al. 2023	1	19	2	17	17.6%	0.45 [0.04, 4.50]	2023
Valorenzos et al. 2025	5	74	8	65	82.4%	0.55 [0.19, 1.59]	2025
**Total (95% CI)**		**93**		**82**	**100.0%**	**0.53 [0.20, 1.39]**	
Total events	6		10				
Heterogeneity: Tau² = 0.00; Chi² = 0.02, df = 1 (P = 0.87); I² = 0%							
Test for overall effect: Z = 1.29 (P = 0.20)							

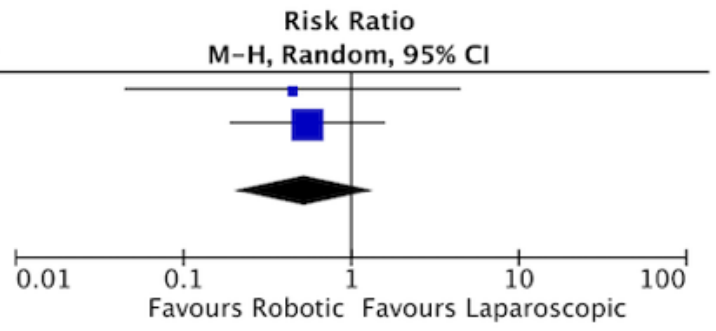

D) Seroma

Study or Subgroup	Robotic Events	Robotic Total	Laparoscopic Events	Laparoscopic Total	Weight	Risk Ratio M-H, Random, 95% CI	Year
Prabhu et al. 2020	6	48	3	54	44.8%	2.25 [0.59, 8.51]	2020
Wikiel et al. 2023	1	19	3	17	28.1%	0.30 [0.03, 2.60]	2023
Valorenzos et al. 2025	1	74	3	65	27.1%	0.29 [0.03, 2.75]	2025
**Total (95% CI)**		**141**		**136**	**100.0%**	**0.73 [0.17, 3.25]**	
Total events	8		9				
Heterogeneity: Tau² = 0.83; Chi² = 3.79, df = 2 (P = 0.15); I² = 47%							
Test for overall effect: Z = 0.41 (P = 0.68)							

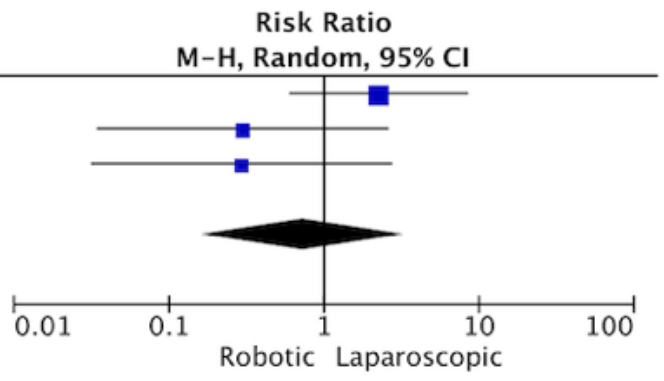

E) Urinary retention

Study or Subgroup	Robotic Events	Robotic Total	Laparoscopic Events	Laparoscopic Total	Weight	Risk Ratio M-H, Random, 95% CI	Year
Prabhu et al. 2020	1	48	1	54	26.9%	1.13 [0.07, 17.50]	2020
Wikiel et al. 2023	1	19	1	17	27.9%	0.89 [0.06, 13.23]	2023
Valorenzos et al. 2025	1	74	7	65	45.1%	0.13 [0.02, 0.99]	2025
**Total (95% CI)**		**141**		**136**	**100.0%**	**0.39 [0.09, 1.71]**	
Total events	3		9				
Heterogeneity: Tau² = 0.14; Chi² = 2.17, df = 2 (P = 0.34); I² = 8%							
Test for overall effect: Z = 1.24 (P = 0.21)							

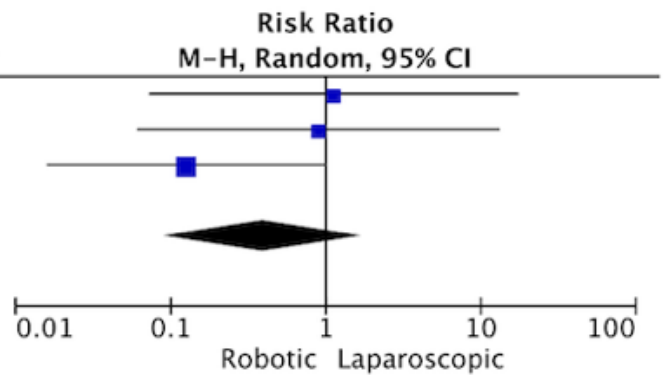

F) Hematoma

Study or Subgroup	Robotic Events	Robotic Total	Laparoscopic Events	Laparoscopic Total	Weight	Risk Ratio M-H, Random, 95% CI	Year
Prabhu et al. 2020	1	48	0	54	26.1%	3.37 [0.14, 80.76]	2020
Valorenzos et al. 2025	5	74	12	65	73.9%	0.37 [0.14, 0.98]	2025
**Total (95% CI)**		**122**		**119**	**100.0%**	**0.65 [0.10, 4.45]**	
Total events	6		12				
Heterogeneity: Tau² = 1.04; Chi² = 1.72, df = 1 (P = 0.19); I² = 42%							
Test for overall effect: Z = 0.43 (P = 0.66)							

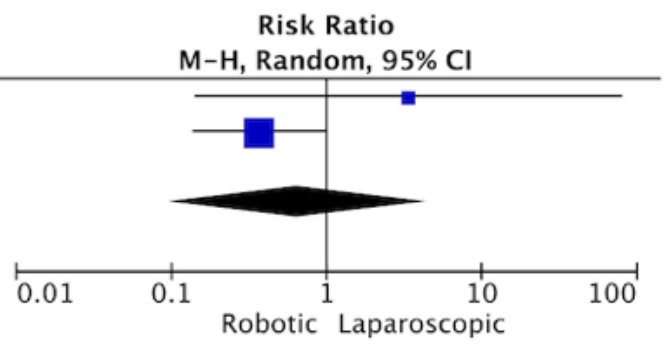

G) Hospital readmission

Study or Subgroup	Robotic Events	Robotic Total	Laparoscopic Events	Laparoscopic Total	Weight	Risk Ratio M-H, Random, 95% CI	Year
Valorenzos et al. 2025	1	74	8	65	60.5%	0.11 [0.01, 0.85]	2025
Dixon et al. 2025	1	39	0	20	39.5%	1.57 [0.07, 37.00]	2025
**Total (95% CI)**		**113**		**85**	**100.0%**	**0.31 [0.02, 4.09]**	
Total events	2		8				
Heterogeneity: Tau² = 1.74; Chi² = 1.94, df = 1 (P = 0.16); I² = 48%							
Test for overall effect: Z = 0.88 (P = 0.38)							

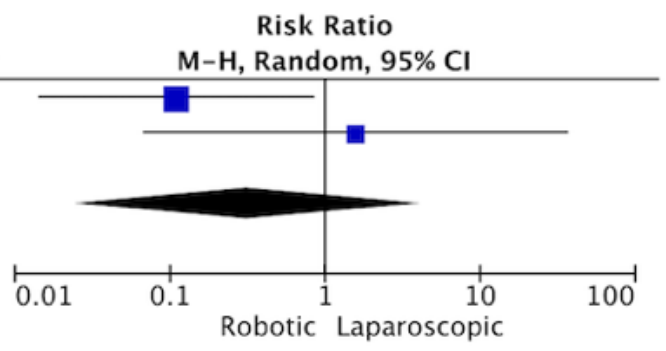

Supplement: Supplementary file 1 [file 464_2026_13253_MOESM1_ESM.pdf]
